# Infant Feeding Challenges in the First Six Months: Influencing Factors, Consequences, and Strategies for Maternal Support

**DOI:** 10.3390/nu17061070

**Published:** 2025-03-19

**Authors:** Karolina Krupa-Kotara, Jarosław Markowski, Mateusz Grajek

**Affiliations:** 1 Department of Epidemiology, Faculty of Public Health in Bytom, Medical University of Silesia, 41-902 Bytom, Poland; 2 Department of Laryngology, Faculty of Medical Sciences, Medical University of Silesia, 40-055 Katowice, Poland; jmarkowski@sum.edu.pl; 3 Department of Public Health, Faculty of Public Health in Bytom, Medical University of Silesia, 41-902 Bytom, Poland; mgrajek@sum.edu.pl

**Keywords:** infant feeding, breastfeeding, formula feeding, mixed feeding, feeding difficulties, maternal education, BMI, Montreal Children’s Hospital Feeding Scale, early childhood nutrition, maternal support strategies

## Abstract

**Introduction:** The first six months of an infant’s life are crucial for the child’s physical and psychological development. During this period, maternal feeding practices significantly impact infant nutrition and growth. The aim of this study was to identify factors associated with feeding difficulties in infants younger than six months. **Methods:** The study was conducted using a CAWI method. The study group consisted of 555 mothers who completed an online questionnaire about demographics, feeding methods, and feeding difficulties experienced, measured using the Montreal Children’s Hospital Feeding Scale (MCH-FS). Infant feeding methods were clearly distinguished as direct breastfeeding, breast milk feeding (expressed milk), and formula feeding. Statistical analyses included effect sizes (Cohen’s d) and 95% confidence intervals (95% CI). **Results:** In the study group, 65% of mothers reported feeding difficulties. Significant predictors of feeding difficulties included maternal overweight and obesity (*p* = 0.041; Cohen’s d = 0.37, 95% CI [0.15, 0.59]), lower maternal education (*p* = 0.014; Cohen’s d = 0.45, 95% CI [0.22, 0.68]), lack of adequate partner support (38%), and the use of mixed feeding methods (mean difference = 4.4 points, *p* = 0.027; Cohen’s d = 0.46, 95% CI [0.23, 0.69]). **Conclusions:** Feeding difficulties during infancy are common and influenced by maternal health and sociodemographic factors. These findings emphasize the importance of targeted educational resources and lactation support interventions tailored specifically to mothers at increased risk.

## 1. Introduction

The first six months of a baby’s life are a critical period for its health and development, during which proper nutrition plays a key role. Breast milk, considered the gold standard of infant nutrition, provides a complete set of essential nutrients, including proteins, fats, carbohydrates, vitamins and minerals, and bioactive factors that support immunity and the maturation of physiological systems [1]. The composition of breast milk is dynamic, adapting to the baby’s changing needs at different stages of development [2]. Leading health organizations, such as the World Health Organization (WHO); the American Academy of Pediatrics (AAP); and the European Society for Pediatric Gastroenterology, Hepatology and Nutrition (ESPGHAN), recommend exclusive breastfeeding for the first six months of life, with continued feeding in combination with complementary foods until at least two years of age [3,4,5]. The latest ESPGHAN guidelines emphasize the importance of a flexible approach to the introduction of complementary foods, tailored to the individual readiness of the child. Although exclusive breastfeeding for the first six months remains recommended, ESPGHAN indicates the possibility of earlier introduction of solid foods in some cases [5].

Alternatively, formula is a safe and widely used nutritional option for infants who are not breastfed. Modern formulas are enriched with prebiotics, probiotics, omega-3 fatty acids, and nucleotides to make their composition close to breast milk [6]. Although feeding with formula is not associated with the technical problems characteristic of breastfeeding, such as nipple soreness or difficulty in achieving a proper latch, it can cause other challenges, such as difficulties in adequately preparing the milk, maintaining bottle hygiene, and ensuring proper formula selection for infants with metabolic or allergy-related conditions [7].

Research indicates that breastfeeding and formula milk feeding can involve challenges related to parent–child interaction, understanding the infant’s needs, and parental concerns about proper nutrition [8]. A parent’s feeding style, defined as the approach and strategy used when feeding a child, can play a key role in developing feeding difficulties. Parents who use a more responsive feeding style that considers their child’s feeding cues show greater effectiveness in preventing feeding problems than parents who use a controlling or restrictive approach [9]. On the other hand, inconsistency in the feeding style used or excessive control can lead to the development of abnormal eating habits in the child [10]. Demographic and social factors, such as the mother’s age, education level, socioeconomic status, and place of residence, can significantly influence infant feeding experience [11].

The six-month feeding period, during which the exclusive use of breast milk or formula is recommended, is also a key point in forming a child’s eating habits. During this time, most parents avoid early dietary expansion, following WHO, AAP, and ESPGHAN recommendations. Only a few parents decide to introduce complementary foods earlier [12]. This makes the first six months of a child’s life a period in which the feeding-related variables analyzed are not affected by additional factors such as dietary diversity. Focusing on this age group allows a more precise study of the relationship between feeding methods, difficulties encountered by parents, and potential risk factors [13].

There is growing interest in the literature in standardized tools for assessing feeding difficulties, which allow a more accurate understanding of mothers’ problems. The Montreal Children’s Hospital Feeding Scale (MCH-FS) is one of the most comprehensive and widely used tools. This scale was designed to assess feeding difficulties in children between the ages of six months and six years, covering aspects such as feeding behavior, oral sensory and motor functions, appetite, parental concerns, strategies used by parents, and family responses to feeding difficulties [14].

Although the original age range of the MCH-FS covers children from six months to six years, there is reason to apply it to younger infants as well. This is due to the universality of the areas assessed, such as feeding behavior and parental responsiveness, which are also important in the first months of a child’s life. Preliminary research indicates that key areas assessed by the MCH-FS, such as parent–child interactions or coping strategies for feeding difficulties, can also be effectively analyzed in infants younger than six months. Validation of the scale in this younger age group may help expand its use and provide a valuable diagnostic tool for early detection of problems and effective intervention [15].

The MCH-FS was originally validated in English in a population of children with and without feeding difficulties and has been translated, adapted, and validated in several languages, including French [14], Thai [16], Portuguese [17], and Dutch [18]. However, Poland lacks a tool of this type, making it difficult for medical professionals to identify and assess feeding difficulties and provide effective support. The introduction of a validated Polish version of the MCH-FS for children in the first six months of life will provide a standardized tool for the rapid detection of feeding problems, enabling the implementation of appropriate interventions.

The decision to focus on infants in the first six months of life is due to the unique characteristics of this period. This is the stage when both breastfeeding and formula play a key role in ensuring optimal child development. Older children typically transition to a complementary diet, which reduces reliance on breast or formula and introduces additional variables into the analysis. In addition, studies indicate that the greatest feeding difficulties occur in the first months of life, making this age group particularly relevant for analyzing risk factors and designing effective interventions [19]. Focusing on this group also enables more precise use of tools such as the MCH-FS, which were developed to assess specific challenges during this crucial period [20,21,22,23].

The purpose of this study is to analyze the most common feeding difficulties for infants in the first six months of life, both breast and formula, and to identify the demographic, socioeconomic, and health factors that influence their occurrence. In addition, the study aims to assess the importance of using a standardized tool, the MCH-FS scale, in monitoring and analyzing feeding difficulties in the first six months of life, which may contribute to the development of more effective maternal support programs. The main outcome variables included the frequency and severity of feeding difficulties in infants; the relationship between the level of difficulty and demographic factors (education, BMI, place of residence) and the comparison of difficulties between feeding methods (breastfeeding, mixed feeding, formula feeding). For clarity and consistency, the following terminology was applied throughout the manuscript: Breastfeeding refers exclusively to direct feeding from the breast, breast milk feeding refers specifically to feeding expressed breast milk from a bottle or other feeding devices, and formula feeding refers to feeding infant formula exclusively or predominantly.

## 2. Materials and Methods

### 2.1. Test Procedure

The study was designed as a cross-sectional one to analyze infant feeding difficulties in the first six months of life. It was conducted between January and May 2024. Participants were recruited using the CAWI (Computer-Assisted Web Interview) method, which allowed for the collection of data in an anonymous manner that was convenient for respondents and reduced potential data entry errors [24,25]. The questionnaire was distributed to parenting groups on social media platforms that brought together mothers of infants of different ages. The recruitment announcement clearly stated the purpose of the study and emphasized the voluntary and anonymous nature of participation. Respondents were informed that they could opt out of completing the survey at any stage without giving a reason. The use of the Facebook platform made it possible to reach a wide range of potential participants, which allowed the study group to be diverse in demographics and socioeconomic background. This cross-sectional design allows for capturing associations at a specific time point but does not establish causal relationships. Future studies should consider a longitudinal approach to assess how feeding difficulties evolve over time. Nevertheless, the present study provides an important reference point for further research.

Considering data from the Central Statistical Office (CSO) relating to the state and structure of the population and natural movement in the territorial section, the minimum sample size for the survey was calculated. According to the CSO report, in the second half of 2023, the number of children up to six months of age was 1,088,411. The minimum sample size was calculated based on the formula [26]:

Nmin =NP − (α^2^ − f(1 − f)) ÷ NP − e^2^ + α^2^) − f(1 − f)

where Nmin—the minimum sample size; NP—the size of the population from which the sample was taken; α—the confidence level for the results; f—the size of the fraction; e—the assumed maximum error. It was determined that under the 95% confidence level, 0.9 fraction size, and 5% maximum error, the minimum sample size was 138 people. The study included 555 women, which significantly exceeded the required minimum sample size, thus increasing the accuracy of the results and their representativeness for the population of mothers of infants in Poland. The minimum sample size was calculated based on key outcome variables such as the frequency of feeding difficulties, their relationship to demographic factors, and a comparison of difficulties between feeding methods. The calculated value allows for statistically significant results in the analyzed groups.

### 2.2. Inclusion Criteria

Women who met strict inclusion criteria, including being at least 18 years old, having a child less than 6 months old at the time of the study, and having no significant chronic conditions or psychiatric disorders that could affect perceived feeding difficulties, were eligible for the study. Women whose children were born prematurely or required specialized feeding interventions were excluded from the study. Respondents who did not fully complete the survey were also excluded. To ensure the study sample consisted of mothers without significant psychiatric conditions that could influence their feeding experience, participants were asked the following screening question: ‘Do you have any diagnosed psychiatric disorders that could affect your experience with infant feeding?’ Respondents who answered ‘yes’ were excluded from the study. This self-reported exclusion criterion was implemented to maintain the focus on healthy mother–infant dyads. Of the initial 612 participants who accessed the questionnaire, 555 completed it fully (90.7% completion rate).

### 2.3. Characteristics of the Study Group

Finally, 555 women between 19 and 46 were eligible for the study. Most participants (57.12%) were in the 26–33 age range. Table 1 and Table 2 present detailed demographic and socioeconomic characteristics of the study group, including variables such as age, number of pregnancies, education level, place of residence, and body mass index.

The largest group among those surveyed were women aged 26–33 (57.12%). As many as 378 (68.12%) of the women in the study group were after their first pregnancy at the time of the survey, while 177 (31.89%) of the women had gone through more than one pregnancy. Overall, 76.6% of the women had a college education, while 20.9% had a high school education. Working women outnumbered other women, and there were 443 (79.8%). Moreover, 368 (66.3%) of the women described their residence as a city, while the rest of the respondents lived in the countryside, which accounted for 33.7%. The body mass index for 59.28% of the women was normal, but the remaining women were underweight (4.86%), overweight (23.24%), or obese (12.61%). Most participants lived in urban areas (66.3%), while 33.7% resided in rural regions. Given the online recruitment method, women with higher socioeconomic status and access to digital resources might be overrepresented. Future studies should incorporate diverse recruitment strategies to ensure the representation of lower-income and non-digital populations.

### 2.4. Research Tool

The proprietary survey questionnaire developed for the project consisted of four main sections designed to comprehensively explore the issue of infant feeding difficulties in the first six months of life. The first section included nine sociodemographic questions to collect data on the mother and child’s age, number of pregnancies, education level, place of residence, and occupational status. The second section included 12 questions about respondents’ breastfeeding knowledge and the difficulties encountered. These questions included the health benefits of breastfeeding for the child and mother, sources of knowledge about breastfeeding, feeding methods used (breast, formula, mixed feeding), and feeding length. The third section of the questionnaire included 10 questions assessing the difficulty level in feeding. Respondents rated difficulties in synchronizing feedings with the day’s schedule, positioning the baby, and soreness during feedings. Responses were rated on a 5-point scale, and the aggregate scores allowed for the classification of difficulties as minimal, moderate, significant, excellent, or very great. The fourth section of the questionnaire included the Montreal Children’s Hospital Feeding Scale, translated into Polish and adapted for the study group. The MCH-FS scale contained 14 questions rated on a 7-point Likert scale on aspects such as the course of feeding, its duration, the child’s appetite, and the parents’ reactions to feeding difficulties. Negatively worded questions were reverse scored to ensure consistency of interpretation. The MCH-FS was translated into Polish following a standardized cross-cultural adaptation process. The translation followed the recommended steps: forward translation, expert panel review, back-translation, and cognitive debriefing with a pilot group of 30 mothers. The tool demonstrated excellent internal consistency (Cronbach’s α = 0.91) and strong test–retest reliability (r = 0.94, *p* < 0.001). A full version of the translated tool is available in the Supplementary Materials to facilitate further research and clinical application.

The tool for assessing the level of difficulty in feeding a child in the first six months of life was validated to confirm its relevance and reliability in the study population. Various statistical methods were applied to a sample of 100 randomly selected participants from the study group. The validation process included several key steps.

The first step was to assess the tool’s internal consistency using the Cronbach coefficient. The α value was 0.91, indicating perfect internal consistency of the questions comprising the scale. This result confirms that the questions in the tool are strongly interrelated and measure a unified construct, such as feeding difficulties.

The next step was to assess the concordance of the test–retest responses. For this purpose, a retest was conducted in a group of 50 female participants two weeks after the first survey was completed. A high correlation coefficient was obtained (r = 0.94, *p* < 0.001), indicating the stability of the tool’s results over time. This means that the results remain consistent and independent of when the survey was conducted.

In addition, the concordance of responses between respondents was assessed using the Kappa index. The result κ = 0.83 (*p* < 0.001) indicated a high degree of concordance between female participants’ reactions, confirming the tool’s reliability in the context of the study group.

Finally, a construct validity analysis was conducted to assess whether the tool measures child feeding difficulties. The scale results correlated with those of the MCH-FS, used in the study as a complementary reference tool. The correlation coefficient was r = 0.88 (*p* < 0.001), proving that the tool effectively reflects feeding difficulties and is accurate for assessing them in a group of infants in the first six months of life (Table 3).

The tool’s validation showed its high relevance and reliability, which confirms its applicability in research and clinical practice to identify infant feeding problems. Thus, the scale could be used to monitor feeding difficulties and design appropriate interventions to support mothers.

### 2.5. Research Ethics

The study was conducted by the principles of the Declaration of Helsinki, ensuring full respect for the rights of female participants. Data were collected and processed anonymously, and only the study’s authors had access to them. Approval for the project was obtained from the Bioethics Committee of the Silesian Medical University in Katowice (Approval No. BNW/NWN/0052/KB/295/23/24, issued on 14 December 2023). Taking the questionnaire was tantamount to consenting to participate in the study.

### 2.6. Statistical Analysis

All collected data were statistically analyzed using the Statistica StatSoft 13.0 Poland package. Before analysis, the data were verified for completeness and correctness of entry. The study included all variables, both quantitative and categorical, included in the questionnaire.

Descriptive analysis was performed for quantitative variables such as age, number of pregnancies, and BMI. Mean values, medians, standard deviations, and ranges (minimum and maximum values) were calculated. The distribution of these variables was verified for normality using the Shapiro–Wilk test to determine whether to use parametric or non-parametric tests for further analysis.

For categorical variables such as education, occupational status, place of residence, and marital status, percentage distributions were analyzed. The Chi-square test (χ^2^) was used for group comparisons to check the relationship between categorical variables. The results were further verified for variables with more than two categories by analyzing numerical distributions.

The Feeding Difficulty Assessment Scale results were analyzed by calculating the mean scores in the different groups and comparing them between groups using the student’s *t*-test for independent samples, or the Mann–Whitney U-test was applied when the data did not follow a normal distribution, as assessed by the Shapiro–Wilk test. The Kruskal–Wallis test was used to analyze variance ANOVA or its non-parametric equivalent for multiple comparisons. In Table 4, we compare the frequency of reported feeding difficulties among participants. The *p*-values were derived from Chi-square (χ^2^) tests to assess statistical significance. 

The survey tool’s validation results were analyzed using the Cronbach coefficient to assess internal consistency. The tool’s stability over time was evaluated using the test–retest correlation coefficient, while the consistency of responses between respondents was verified using the Kappa index. The tool’s accuracy was confirmed by correlation analysis of MCH-FS scale scores.

The level of statistical significance in all analyses was set at *p* ≤ 0.05. All results are presented as means with standard deviations for quantitative variables and counts and percentages for categorical variables. Where necessary, confidence intervals at the 95% level are also presented.

## 3. Results

The most common infant feeding difficulty reported by mothers during the first six months of life was difficulty properly latching the baby to the breast, affecting 240 women, or 53.7% of the survey participants. Soreness of the nipples was the second most common problem, reported by 238 women, or 53.2% of the survey participants. Irritability of the baby was a problem for 178 mothers, accounting for 39.7%, while maternal fatigue was reported by 169 women or 37.7% of respondents. Further difficulties, such as too little breastmilk and the feeling of not having enough breastmilk, were reported by 151 (33.7%) and 122 (27.2%) of the survey participants, respectively. Less common, though still significant, problems were mastitis, which affected 70 mothers (15.5%), and a lack of or weak suckling reflex, reported by 64 women (14.2%). Statistical analyses showed that most of the reported problems, such as difficulty in achieving a proper latch, nipple soreness, baby irritability, and maternal fatigue, were statistically significant, underscoring the importance of these difficulties in the daily experience of nursing mothers. Some less common problems, such as mastitis and lack of suckling reflex, did not reach statistical significance, but their impact on comfort and feeding efficiency cannot be ignored. These findings underscore the need for targeted support for mothers, particularly in the areas of child attachment techniques and reducing nursing fatigue and pain (Table 4). Mothers practicing mixed feeding reported significantly greater feeding difficulties compared to exclusive breastfeeding mothers (*p* = 0.003, Cohen’s d = 0.45, 95% CI [0.21–0.69]).

### 3.1. Distribution of Difficulty Levels in Infant Feeding

The analysis assessed the level of difficulty in feeding infants during the first six months of life. In the study group of women, a moderate level of difficulty was most often reported (35%), while significant difficulties were reported by 30% of mothers. Minimal difficulties were present in 15% of the respondents, while 15% reported great difficulties, and 5% reported very great difficulties (Table 5).

### 3.2. Relationship of Difficulty Level to Mother’s Education Level

The Chi-square test showed a statistically significant relationship between the level of education and the category of difficulty in feeding the child (*p* = 0.014). Mothers with higher education most often reported minimal difficulties (20%) and rarely reported very great difficulties (4%). In contrast, mothers with primary education more often experienced significant difficulties (40%) and very great difficulties (10%) (Table 6).

### 3.3. Relationship of Feeding Method to Level of Difficulty

The analysis of differences in difficulty level by feeding method was conducted using ANOVA and the Kruskal–Wallis test. Both tests showed significant differences between the groups (*p* = 0.027 *p* = 0.027 *p* = 0.027 for ANOVA, *p* = 0.032 *p* = 0.032 *p* = 0.032 for Kruskal–Wallis). Mothers using mixed feeding experienced the greatest difficulties (mean 30.2), while the lowest level of difficulties was reported by mothers exclusively breastfeeding (mean 25.8) (Table 7). In Table 7 and Table 8, we conducted post hoc analyses to further investigate group differences: the Dunn–Bonferroni test was applied following the Kruskal–Wallis test, and the Tukey post hoc test was used after ANOVA to identify specific pairwise differences between groups.

### 3.4. The Impact of BMI on the Level of Difficulty

Analysis of the effect of BMI on the level of difficulty in feeding showed that underweight mothers (BMI < 18.5) reported relatively low levels of difficulty (M = 23.8), while the highest difficulties were in obese mothers (M = 30.1). The use of the Kruskal–Wallis test revealed a significant difference between BMI groups (*p* = 0.041) (Table 8).

Mothers with higher education experienced significantly lower feeding difficulties than mothers with primary and secondary education (B = −2.15; *p* = 0.004; Cohen’s d = 0.45, 95% CI [−0.68, −0.22]). Mothers using formula milk feeding and mixed feeding reported significantly higher difficulty levels than mothers exclusively breastfeeding (B = 3.25, *p* < 0.001; Cohen’s d = 0.46, 95% CI [0.23, 0.69]). Overweight and obese mothers reported significantly higher feeding difficulties compared to mothers with normal BMI (B = 3.15; *p* = 0.002; Cohen’s d = 0.37, 95% CI [0.15, 0.59]). Analyses revealed that women living in rural areas reported significantly higher feeding difficulties than those in urban areas (B = 2.92, *p* = 0.027; Cohen’s d = 0.29, 95% CI [0.07, 0.52]). Furthermore, mothers who received lower levels of partner support experienced significantly higher feeding difficulties (B = 3.44, *p* = 0.021; Cohen’s d = 0.34, 95% CI [0.12, 0.56]) (Table 9).

Statistical analyses revealed significant correlations between infant feeding difficulties and key demographic and social factors, such as the mother’s education, feeding method, and BMI. The results indicate the need to tailor support strategies to the specific needs of mothers, especially those with higher levels of feeding difficulties. Mothers using mixed feeding and women with higher BMI values are groups that require special attention and appropriate support.

## 4. Discussion

Difficulties in feeding infants in the first six months of life are a global problem that affect the health of both mother and child. In our study, 83.5% of mothers reported difficulties, indicating the need for early identification and intervention. Similar results were reported in a survey by Gianni et al. [27,28], who found that 70–80% of women experienced breastfeeding difficulties, such as nipple pain, feeling inadequate, or fatigue. The discrepancy in partner support observed in our study (38% lacking support) compared to Gianni et al. (12%) might be influenced by cultural and societal differences, varying family structures (extended versus nuclear families), and differing expectations regarding parental roles. In cultures or social environments where extended family involvement is less common, mothers may perceive lower levels of direct partner support, impacting feeding practices and maternal confidence. Our study’s high percentage of difficulties may be due to a detailed assessment tool, the MCH-FS, which allows for a comprehensive analysis of feeding problems or difficulties.

The recruitment of participants for our study was carried out in a way that enabled us to reach a wide range of mothers with different experiences of infant feeding. Participants were recruited through parenting communities on online platforms, which allowed us to access mothers with different levels of education, economic statuses, and places of residence. To assess potential selection bias, we compared our sample with population data from the Central Statistical Office (GUS) and reports on breastfeeding practices in Poland. The data from the Central Statistical Office (GUS) [29] indicate that the percentage of infants exclusively breastfed in the first month of life is around 86% and drops to 55% after six months. In our sample, 68% of mothers declared initial breastfeeding, and 35% continued it as the exclusive method until six months, which is in line with national trends. In the study, we considered a wide spectrum of difficulties related to breastfeeding, such as problems with latching the baby to the breast (53.7% of mothers), sore nipples (53.2%), feeling like there is not enough milk (33.7%), tiredness related to feeding (37.7%), and mastitis (15.5%). Taking these variables into account allows for a comprehensive analysis of feeding difficulties in a way that considers the actual background population. In addition, clearly defined inclusion and exclusion criteria for participants minimized the impact of extreme cases (e.g., prematurity, serious maternal and child conditions). Our analysis of the outcome variables and their statistical relationships required a sample size that exceeded the minimum calculation, which allowed for more detailed comparisons between groups and increased the statistical power of the results.

Our results indicate that women with higher education and living in urban areas were less likely to report feeding difficulties. According to the literature, higher education is a protective factor, supported by a study by Ramsay et al. [14], who emphasized that education influences greater awareness of the benefits of breastfeeding and access to educational resources. Ren et al. [30] also indicated that better accessibility to specialized medical care in urban areas may minimize breastfeeding difficulties. This may be due to greater access to educational resources, professional lactation support, and increased awareness of breastfeeding benefits. Additionally, higher-educated mothers may be more confident in seeking medical advice or using problem-solving strategies. Future studies should explore whether educational interventions targeted at lower-educated mothers could mitigate feeding difficulties.

In our study, mothers using mixed feeding reported the highest level of difficulty, which may be related to the need to manage different feeding methods and the logistics of preparing formulas. Studies by Gianni et al. [27,28] and Komninious et al. [31] indicated that mixed feeding is more burdensome for mothers regarding time and emotion. The high percentage of mothers choosing mixed feeding or formula milk in our study may have been related to inadequate lactation support in the first days after birth, as suggested by the analysis of Kam et al. [32] and studies conducted in the US [33]. The combination of breastfeeding and formula feeding can introduce additional challenges, such as nipple confusion, reduced milk supply due to inconsistent breastfeeding, and stress associated with balancing different feeding methods. A more detailed exploration of maternal experiences with mixed feeding could provide insight into how to better support this growing group of mothers.

The results of our study indicate that difficulties with breastfeeding may influence mothers’ decisions to introduce solid foods earlier. This is in line with current ESPGHAN guidelines [5], which suggest an individual approach to expanding infants’ diets, considering factors such as lactation difficulties and the child’s nutritional needs. ESPGHAN’s flexible guidelines on the introduction of complementary feeding suggest variability in timing and approach, which our findings indicate might contribute to increased feeding difficulties. Early introduction of complementary foods could potentially exacerbate feeding issues, highlighting the importance of consistent, evidence-based guidance and professional support to prevent or mitigate feeding challenges.

The effect of maternal weight on feeding difficulties is an important finding of our study. Overweight and obese women were significantly more likely to report feeding difficulties. These findings align with the survey by Perz et al. [34], who showed that women with excessive body weight have more significant breastfeeding difficulties due to baby positioning problems and delayed milk let-downs. In addition, Martin et al. [35] indicated that excessive weight gain during pregnancy was associated with early cessation of breastfeeding. Therefore, education and support for women with an abnormal BMI should be an integral part of perinatal care. Overweight and obese mothers may experience increased feeding difficulties due to physiological factors, such as delayed lactogenesis II, reduced prolactin response, or mechanical difficulties in positioning the infant. Psychological factors, including body image concerns and stress, may also contribute. Future research should investigate these mechanisms in more detail to tailor support programs accordingly.

Lack of emotional support from the partner and family is a significant factor hindering breastfeeding. In our study, 38% of women indicated a lack of support from their partner, which is significantly higher than in Gianni et al. [27,28], where only 12% of mothers could count on help from loved ones. Emotional support plays a key role in preventing postpartum depression, which can affect the decision to continue breastfeeding. Studies by Rozensztrauch et al. [36], Cohen [37], and Szymkowiak et al. [38] emphasized that adequate support in the first months of a child’s life reduces the risk of postpartum depression and supports breastfeeding maintenance.

In our study, women who had a Cesarean section were more likely to report feeding difficulties than women giving birth naturally. These findings are consistent with the study by Hobbs et al. [39], which indicated that women who had had a Cesarean section experienced greater difficulty in latching on to the breast and delayed milk infusion. Additional lactation support for this group of women is crucial to minimize the difficulties experienced and increase the percentage of mothers who breastfeed.

The use of the MCH-FS scale in our study made it possible to accurately identify the level of feeding difficulties and analyze their relationship with sociodemographic and health factors. The high accuracy (r = 0.88) and reliability (κ = 0.83) of the tool confirm its value in scientific research and clinical practice. The tool can also be used effectively with infants in the first 6 months of life, although it was originally designed for children aged 6 months–6 years.

Our study provides important data on infant feeding difficulties and the factors determining them. The results indicate the need for integrated education, emotional support, and lactation interventions, especially for groups of mothers at the highest risk of difficulties.

### 4.1. Strengths and Limitations of the Study

The study of infant feeding difficulties in the first six months of life stands out for several important strengths. First and foremost, the representative sample of 555 women allows conclusions to be drawn with high reliability and potential application to the wider population. The diversity of the sample, which included women of different ages, education levels, body mass indices (BMI), and occupational statuses, enabled a comprehensive analysis of the impact of sociodemographic factors on feeding difficulties.

The questionnaire used in the study was comprehensively prepared, considering both technical aspects of feeding and emotional and social factors. The use of the Montreal Children’s Hospital Feeding Scale (MCH-FS), which was validated in the study group, increased the quality of the data obtained. The Cronbach coefficient value (α = 0.91) and high accuracy (r = 0.88) confirm that the tool effectively measures feeding difficulties in this age group.

The primary limitation of this study is the reliance on self-reported data obtained exclusively through an online survey (CAWI), which may introduce selection and reporting biases. Specifically, this recruitment strategy might disproportionately represent mothers with greater internet access and higher socioeconomic status, potentially limiting the generalizability of the findings. Self-reporting methods, despite assurances of anonymity, could lead to inaccuracies stemming from recall errors, subjective interpretations, and tendencies toward socially desirable responses. Consequently, these factors may have impacted the accuracy, and prevalence estimates of reported feeding difficulties. Future research should employ more diversified recruitment methods to include a broader demographic spectrum and incorporate objective assessments, such as clinical evaluations or structured observations, to validate and strengthen these findings. Additionally, adopting a longitudinal approach would facilitate better understanding of causal relationships and the evolution of feeding difficulties over time.

The study sample included 555 women with varying educational backgrounds, BMI categories, and places of residence. However, 76.6% of participants had a higher education, which is above the national average. This suggests that the sample may overrepresent mothers with greater health awareness or access to breastfeeding education. Although the study aimed to recruit a diverse sample, the findings should be interpreted with this limitation in mind. Future research should include additional recruitment strategies to ensure broader representation of lower-educated and socioeconomically disadvantaged mothers.

In addition, the survey covers only the first period of a child’s life, which does not allow an assessment of the long-term effects of feeding difficulties. The high percentage of mothers with a college education (76.6%) may also introduce some distortion, as this group may be more aware of the benefits of breastfeeding. The lack of consideration of other important variables, such as the mothers’ mental health status or level of social support, is another aspect that needs to be developed in future studies.

This study provides reference data for feeding difficulties in healthy mother–infant dyads. This is crucial as most research focuses on high-risk groups, while our findings indicate that a substantial proportion of mothers in the general population also experience moderate-to-severe feeding difficulties. These reference values may facilitate comparisons with populations facing additional medical or socioeconomic challenges, allowing for a more targeted approach in future interventions.

### 4.2. Further Research Directions

Subsequent studies should cover a period longer than the first six months of a child’s life to allow analysis of the long-term consequences of feeding difficulties, such as the impact on child development or continued feeding. An important area is also to examine the role of emotional and social support for mothers, considering the influence of the partner, family, and lactation consultants. It is worth considering enriching research methods with independent observations, interviews with medical professionals, and objective indicators such as clinical data or biomedical analyses. Such an approach would better validate reported difficulties and provide a more complete picture of the problem. Triangulation of methods could increase the reliability and precision of the results, as well as contribute to a deeper understanding of the complexity of feeding difficulties.

An interesting area of research would be to compare the effectiveness of different educational strategies, such as lactation workshops, one-on-one counseling, or group programs, in reducing feeding difficulties. It would also be worth including qualitative research, such as in-depth interviews or focus groups, to obtain a more nuanced picture of mothers’ experiences.

Cultural and socioeconomic context is another important area of research. Understanding how different communities cope with feeding difficulties and what support strategies are most effective in different settings can contribute to more personalized and effective interventions.

Our findings highlight that 65% of mothers reported at least moderate feeding difficulties, which underscores the need for improved support strategies. While high-risk groups often receive targeted interventions, our data suggest that even in a healthy population, feeding difficulties are prevalent and should not be overlooked in public health and lactation support programs.

### 4.3. Practical Implications

The study’s findings have important practical implications for health care and support activities for nursing mothers. First and foremost, they underscore the need to develop systemic educational programs targeting women as early as during pregnancy. These programs should address the diverse needs of mothers, tailoring the content to their education, age, and parenting experience. Given the strong associations between maternal education, BMI, and feeding difficulties, tailored interventions should be implemented. Specific recommendations include:

- Education programs for mothers with lower education levels, focusing on practical breastfeeding techniques and problem-solving strategies.
- Targeted lactation support for overweight and obese mothers, including personalized counseling and early postpartum lactation monitoring.
- Improved access to lactation support in rural areas, through telehealth consultations and community-based peer support groups.

Support for post-Cesarean-section women, who were more likely to report feeding difficulties, should be a priority. Training medical personnel, including midwives and lactation consultants, to deal with the most common problems, such as nipple pain and delayed milk let-down, could significantly improve breastfeeding rates.

Equalizing access to lactation support at the system level is necessary, especially in smaller towns and villages where access to specialists is limited. The development of emotional support programs for mothers, including psychological consultations and support groups, can help reduce the percentage of mothers who give up breastfeeding.

The results of the survey also relate to the implementation of the Organizational Standard of Perinatal Care in Poland, which provides for activities such as kangarooing, rooming-in, and lactation support. Kangarooing, or direct skin-to-skin contact between mother and child immediately after birth, has a significant impact on the initiation of lactation and bonding. Promoting this element can increase the percentage of mothers who breastfeed in the first few days after birth, which has been confirmed in studies showing better lactation outcomes in women who kangarooed their babies.

Rooming-in, the sharing of mother and baby in the same room, promotes feeding on demand, which promotes lactation stimulation and reduces the risk of feeding difficulties. The study’s findings underscore the importance of this element in promoting exclusive breastfeeding, especially in the first days after birth. At the same time, it is crucial to ensure continued access to qualified medical personnel to help mothers cope with technical and emotional problems.

Lactation support, an integral part of the Organizational Standard of Perinatal Care, should be particularly targeted at mothers from at-risk groups, such as post-C-section women or overweight mothers. Training programs for medical personnel, including practical solutions to the most common lactation problems, could significantly improve breastfeeding rates in these groups.

Integration of the research findings with the practice of implementing the Organizational Standard of Perinatal Care and the development of educational programs, emotional support, and lactation consultations can contribute to reducing the percentage of mothers reporting feeding difficulties and increasing the rate of exclusive breastfeeding in the first six months of a child’s life. These proposals are part of the implementation of the recommendations of the World Health Organization (WHO) and the European Society for Pediatric Gastroenterology, Hepatology and Nutrition (ESPGHAN), promoting maternal and child health and improving the quality of perinatal care in Poland.

## 5. Conclusions

Based on the study’s results, it can be concluded that difficulties in feeding infants in the first six months of life are common, especially at moderate and significant levels. Higher levels of maternal education proved to be a protective factor, significantly reducing reported difficulties, while mothers with lower levels of education were more likely to experience challenges. The feeding method also significantly impacted the level of difficulties—mothers using mixed feeding and formula milk reported more difficulties than mothers exclusively breastfeeding. In addition, overweight and obese mothers were associated with higher levels of feeding difficulty, indicating the need for exceptional support for this group. Validation of the MCH-FS tool confirmed its usefulness in assessing feeding difficulties, enabling the identification of groups in need of intervention. The results underscore the need to develop educational and practical support programs for mothers, especially those encountering more significant infant feeding challenges.

## Figures and Tables

**Table 1:** Characteristics of the study group (*n* = 555).

Age	N (%)	M	Me	SD	IQR
19–25 26–33 34–46	56 (10.09%) 317 (57.12%) 182 (32.79%)	30.8	31	4.1	19–46
Number of pregnancies					
First Next	378 (68.12%) 177 (31.89%)	1.3	1	0.6	1–4
Mother’s current BMI					
Underweight Proper body weight Overweight Obesity	27 (4.86%) 329 (59.28%) 129 (23.24%) 70 (12.61%)	24.6	23.8	4.1	18.2–37.5

M—mean; Me—median; SD—standard deviation; IQR—interquartile range.

**Table 2:** Characteristics, distribution, and dominant for categorical variables of the study group (*n* = 555).

Category	Category Value	*n*	%	Dominant
Professional status	Student/Student	13	2.3	Working person
	Working person	443	79.8	
	Non-working person	99	17.8	
Marital status	Miss	13	2.3	In a marriage relationship
	In an informal relationship	120	21.6	
	In a marital relationship	418	75.3	
	Divorcee	3	0.5	
	Widow	1	0.2	
Education	Basic	3	0.5	Higher
	Professional	11	2.0	
	Medium	116	20.9	
	Higher	425	76.6	

**Table 3:** Validation results of the infant feeding difficulty assessment tool.

Validation Criterion	Method	Result	Interpretation
Internal consistency	Cronbach coefficient	α = 0.91	High internal consistency questions in the tool measure a unified construct.
Stability over time	Test–retest	r = 0.94	The tool’s results are highly stable over time and independent of when the test is conducted.
Concordance of responses	Kappa indicator	κ = 0.83; *p* < 0.001	There is a high concordance of responses among respondents; the tool is reliable in the surveyed population.
Relevance of the construct	Correlation with MCH-FS	r = 0.88; *p* < 0.001	The tool effectively measures feeding difficulties and high compliance with a reference tool.

**Table 4:** Most reported difficulties in infant feeding.

Most Commonly Reported Difficulties	*n* (%)	*p*-Value
The problem with properly breastfeeding your baby	240 (53.7%)	<0.001
Soreness of the nipples	238 (53.2%)	<0.001
Child’s irritability	178 (39.7%)	<0.01
Mother’s fatigue	169 (37.7%)	<0.01
Too little food	151 (33.7%)	<0.05
The sensation of lack of food	122 (27.2%)	<0.05
Inflammation of the mammary gland	70 (15.5%)	>0.05
Absence or weak sucking reflex	64 (14.2%)	>0.05

**Table 5:** Distribution of levels of difficulty in feeding infants.

Level of Difficulty	N (%)
Minimal	83 (15.0)
Moderate	194 (35.0)
Significant	166 (30.0)
High	82 (15.0)
Very High	28 (5.0)

**Table 6:** Relationship of education level to categories of feeding difficulties (Chi-square test).

Education	Minimal (%)	Moderate (%)	Significant (%)	High (%)	Very High (%)	χ^2^ *p*-Value
Primary	10.0	25.0	40.0	15.0	10.0	0.014
Vocational	12.0	30.0	35.0	20.0	3.0	
Secondary	18.0	40.0	25.0	12.0	5.0	
Higher	20.0	38.0	28.0	10.0	4.0	

**Table 7:** Level of difficulty according to feeding method (ANOVA and Kruskal–Wallis test).

Feeding Method	M	SD	Me	ANOVA *p*-Value	Kruskal *p*-Value
Breastfeeding	25.8	8.3	24	0.027	0.032
Formula	28.5	9.1	27		
Mixed	30.2	10.2	29		

M—mean; Me—median; SD—standard deviation; *p*-value.

**Table 8:** Level of difficulty according to mothers’ BMI (Kruskal–Wallis Test).

BMI Category	M	SD	Me	Kruskal *p*-Value
Underweight	23.8	7.5	23	0.041
Normal	25.9	8.1	25	
Overweight	28.5	9.2	27	
Obese	30.1	10.4	30	

M—mean; Me—median; SD—standard deviation; *p*-value.

**Table 9:** Identified factors associated with feeding difficulties.

Variable	B	SE	*p*-Value	Cohen’s d	95% CI
Higher education	−2.15	0.78	0.004	0.45	[−0.68, −0.22]
Formula/mixed feeding	3.25	0.85	<0.001	0.46	[0.23, 0.69]
Overweight/obesity (higher BMI)	3.15	0.91	0.002	0.37	[0.15, 0.59]
Rural area	2.92	0.65	0.027	0.29	[0.07, 0.52]
Lower partner support	3.44	0.81	0.021	0.34	[0.12, 0.56]

B—regression coefficient; SE—standard error.

## Data Availability

The original contributions presented in the study are included in the article/Supplementary Materials, further inquiries can be directed to the corresponding author.

## Supplementary Materials

The following supporting information can be downloaded at: https://www.mdpi.com/article/10.3390/nu17061070/s1, Questionnaire.
