# Supplementary material for: Infant Feeding Challenges in the First Six Months: Influencing Factors, Consequences, and Strategies for Maternal Support
Source: Nutrients. 2025 Mar 19;17(6):1070. doi: 10.3390/nu17061070 (PMC11945421; doi:10.3390/nu17061070)
Supplement: Supplementary file 1 [file nutrients-17-01070-s001.zip › nutrients-3535793-supplementary.pdf]

Questionnaire

Dear Madam,

I kindly request your permission to participate in the following study.

The study's purpose is to assess feeding difficulties in children during the first six months of life.

Participation in the survey is entirely voluntary. The whole thing will take about 5 minutes.

I assure you that the study is conducted anonymously and will be used only for scientific purposes. The results will be considered only for scientific purposes—at the level of the study group, not individually.

I strongly urge you to answer the questionnaire honestly. Please mark the answer of your choice in the space provided. Answers are single choice unless the question asks you to mark more than one answer.

I give my informed consent to participate in the study.

Yes

Mother's year of birth

....

Number of pregnancies

....

Mother's height (cm)

....

Mother's current weight (kg)

....

Child's age (weeks)

....

Mother's education:

(a) Elementary

b) Vocational

c) Secondary

d) Higher

What is your professional status?

a) Student/student

b) Working person

c) Non-working person

What is your social status?

a) Miss

b) In an informal relationship

c) Married

d) Divorced

e) Widowed

Place of residence:

(a) Rural

b) City

1) What type of delivery was carried out in your last pregnancy:

(a) Natural childbirth

b) Caesarean section

2) What method of feeding your child did you use?

a) Breastfeeding

b) Feeding with modified milk

(c) Breastfeeding and feeding with modified milk

3) Were there any difficulties during breastfeeding? (If you did not breastfeed, please go to question 6)

a) Yes

b) No

4. did you stop breastfeeding because of the feeding problems you experienced?

a) Yes

b) No

c) Not applicable

5. what difficulties did you experience during breastfeeding? (If you marked the answer “No” in the previous question, please go to the next question)

a) Soreness of the nipples

b) Inflammation of the mammary gland

(c) Problem with properly attaching the baby to the breast

d) Fatigue of the mother

e) Too little milk

f) Irritability of the child

g) Perception of lack of food

h) Defects in the baby's mouth (e.g. cleft lip)

i) Absence or weak sucking reflex

j) Poor condition of the child (e.g. congenital heart defect)

k) Obesity in the mother

l) Medical contraindications to breastfeeding

6) How long do you plan to breastfeed?

a) 3 months

b) 6 months

c) 1 year

d) 2 years

e) I do not intend to breastfeed

7. What sources of knowledge regarding feeding your child have you used?

a) Midwife

b) Childbirth school

c) Nutritionist

d) Internet

8) How would you rate your knowledge of breastfeeding before the birth of your child?

a) Sufficient

b) Insufficient

c) I had no knowledge on the subject

9) Do you know the benefits of breastfeeding for the child and mother

a) Yes

b) No

10) What are the benefits of breastfeeding that may favor the child?

a) Reducing the risk of overweight and obesity

b) Reduced risk of type 1 and type 2 diabetes

c) Rarer occurrence of respiratory infections

d) Reduce the risk of allergies

e) Prevention of malocclusion

f) Development of speech

g) All of the above

h) None of the above

i) Don't know

11) What benefits of breastfeeding may favor the mother?

a) Reduced risk of postpartum hemorrhage

b) Rarer incidence of postpartum depression

c) Reduced risk of breast and ovarian cancer

d) Reduction in the incidence of hypertension

e) Reduction in the incidence of diabetes

f) Faster weight regain

g) All of the above

h) None of the above

i) Don't know

12) Does your partner get involved and help with feeding?

a) Yes

b) No

Baby Feeding Difficulty Rating Scale for the first six months of life (1-5)

Please rate the degree of difficulty you experienced in feeding your baby in the first 6 months of life using the following scale, where:

- None Difficulty: Feeding your baby is going smoothly and efficiently.

- Very Little Difficulty: Slight difficulties that do not cause significant problems during feeding.

- Medium Difficulties: Difficulties occur periodically and require extra attention and effort.

- Major Difficulties: Difficulties are significant and make feeding significantly more difficult.

- Very Large Difficulties: Difficulties are significant and often lead to frustration during feeding.

Please select the number that corresponds to the degree of difficulty you experience when feeding your child:

- Difficulty in recognizing your child's hunger and satiety signals: [ ] (1-5)

- Difficulty in adjusting the child's correct position during feeding: [ ] (1-5)

- Difficulty with painful feeding (e.g., mastitis, sore nipples): [ ](1-5)

- Difficulty understanding the amount of food the baby should eat: [ ] (1-5)

- Difficulty in maintaining regularity of feeding: [ ] (1-5)

- Difficulty in adjusting the diet according to the child's needs: [ ] (1-5)

- Difficulty related to the child's emotional reactions during feeding: [ ] (1-5)

- Difficulty synchronizing the feeding schedule with other responsibilities: [ ] (1-5)

- Difficulty in dealing with possible health problems of the child affecting feeding: [ ] (1-5)

- Difficulty coordinating feeding with caring for other children or household duties: [ ] (1-5)
